# A Stereoscopic Perspective on the Triple-Phase Interface Microenvironment in Electrochemical CO_2_ Reduction: Insights from In Situ Studies

**DOI:** 10.1007/s40820-026-02226-4

**Published:** 2026-05-19

**Authors:** Guiru Zhang, Peng Shen, Xiangrui Li, Lei Zhu, Shuiyun Shen, Junliang Zhang, Zhen Huang

**Affiliations:** 1 Institute of Fuel Cells, School of Mechanical Engineering, Shanghai Jiao Tong University, Shanghai, 200240 People’s Republic of China; 2 Key Laboratory for Power Machinery and Engineering of Ministry of Education, Shanghai Jiao Tong University, Shanghai, 200240 People’s Republic of China

**Keywords:** Carbon dioxide reduction, In situ characterization, Interface spectroscopy, Reaction mechanism, Microenvironment, Structural evolution

## Abstract

When powered by renewable electricity, the electrochemical carbon dioxide reduction reaction (CO_2_RR) represents a transformative strategy for achieving sustainable carbon–neutral cycles. Researchers are striving to decode its ambiguous mechanisms through in situ characterization and establish structure–activity correlations in CO_2_RR systems under operating conditions. Despite these efforts, building a coherent framework that connects insights into atomic-level processes to macroscopic system behavior remains challenging. From this perspective, we present a holistic overview of the critical microregions in CO_2_RR systems and the associated phenomena requiring scrutiny at the triple-phase interface from a stereoscopic perspective. We systematically summarize recent advances in in situ characterization techniques applied across the distinct microregions, including the electrolyte microenvironment, reaction interface, electrocatalyst structure, and diffusion field. By integrating these insights, we highlight advances in mapping the dynamic cross-compartment interactions and transient species formation mechanisms within the CO_2_RR process. Finally, we conclude with suggestions for future research, emphasizing the need for synergistic integration of in situ characterization tools and innovative methodologies to resolve lingering mechanistic ambiguities. Such advancements could accelerate the rational design of efficient CO_2_RR systems and development of sustainable energy solutions.

## Highlights

- The recognition and characterization methods of each microregion at the typical triple-phase interface of carbon dioxide reduction reaction are summarized and elucidated to enhance the understanding of the complex methodologies for interfacial observation and the underlying reaction mechanisms.
- The key species characteristics, electrochemical reaction mechanisms, and core interactions of different microregions are systematically summarized.
- Preliminary explorations of multiple synergistic characterization approaches and potential future in situ characterization strategies are pointed out.

## Graphical abstract

**Figure:**
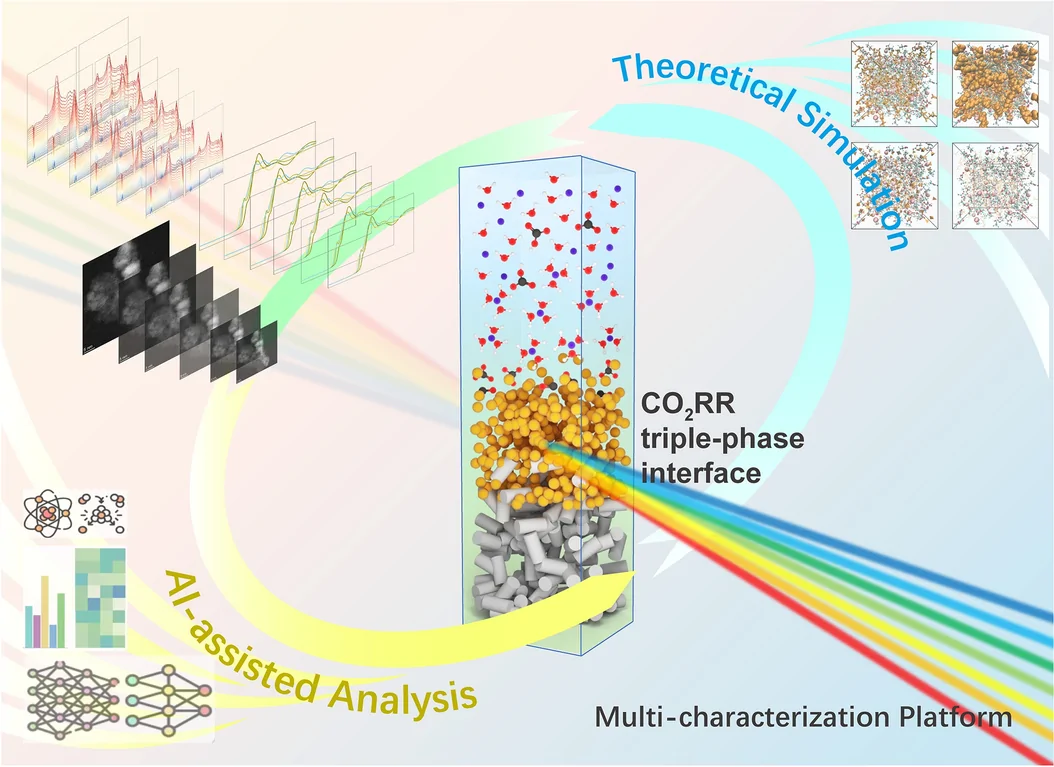

## Introduction

The electrochemical CO_2_ reduction reaction (CO_2_RR) represents a promising strategy for achieving carbon neutrality and sustainable energy storage when powered by renewable energy [1–4]. Since the pioneering work performed by Hori et al. [5, 6], extensive efforts have focused on improving the economic feasibility of the CO_2_RR process [7–9]. Nevertheless, the mechanisms of CO_2_RR remain poorly understood owing to the diversity of electrocatalysts [10, 11], intermediates [12], and products [13], which hampers the elucidation of clear reaction pathways. Additionally, the dynamic evolution of active sites under operating conditions complicates mechanistic interpretations of the structure–activity relationships in CO_2_RR systems, while the inherent complexity of the triple-phase interface poses challenges for real-time monitoring [14–16].

Recent advancements in in situ characterization techniques can provide critical insights to address the aforementioned challenges [17, 18]. These noninvasive approaches enable real-time observations of electrochemical processes, ensuring data reliability. For instance, infrared and Raman spectroscopy have been proven to effectively probe intermediate adsorption and reaction mechanisms [19–22], while synchrotron X-ray techniques (e.g., diffraction and absorption) can facilitate tracking of electrocatalyst structural evolution. Moreover, complementary electron microscopy approaches can resolve nanoscale interfacial morphological changes [23, 24]. Such technological progress has substantially accelerated CO_2_RR research, underscoring the importance of tailored in situ electrochemical characterization for establishing robust structure–performance correlations and guiding the design of highly efficient electrocatalysts.

From a three-dimensional (3D) perspective, the typical gas diffusion electrode (GDE) utilized in CO_2_RR systems can be divided into distinct microregions that undergo continuous dynamic evolution under operating conditions. While in situ characterization techniques enable a multi-perspective examination of overlapping regions, interpreting the data is inherently challenging owing to divergent physicochemical states across microregions [25]. Furthermore, despite substantial efforts to categorize observations from a technical standpoint [26], existing approaches remain inadequate for establishing cross-microregion understanding. We posit that systematic analysis of the interplay between regionalized properties and operational parameters, rather than mere methodological knowledge, is essential for reconciling conflicting observations and advancing mechanistic comprehension.

Herein, we propose a research framework for electrocatalytic interface studies. This framework adopts a multidimensional stereoscopic perspective—integrating spatial (microregions), temporal (dynamic evolution), and multitechnique (combinatorial characterization) dimensions—to provide a systematic review of the triple-phase interfaces in CO_2_RR. Specifically, from a spatial standpoint, the CO_2_RR microenvironment is divided into four microregions: (1) the electrolyte environment, (2) the reaction interface, (3) the electrocatalyst structure, and (4) the diffusion field. Technically, it discusses key topics within each microregion through the synergy of multiple characterization techniques. Temporally, it captures dynamic processes such as ion migration, reaction mechanisms, species evolution, and device operating states within these microregions. Finally, we summarize current knowledge gaps and technical challenges while proposing potential strategies to optimize in situ characterization approaches. These developments could facilitate a comprehensive understanding of the dynamic interfaces in CO_2_RR systems and inform the design of next-generation electrocatalysts.

## Microregions of the Local Reaction Environment

**Scheme 1:**
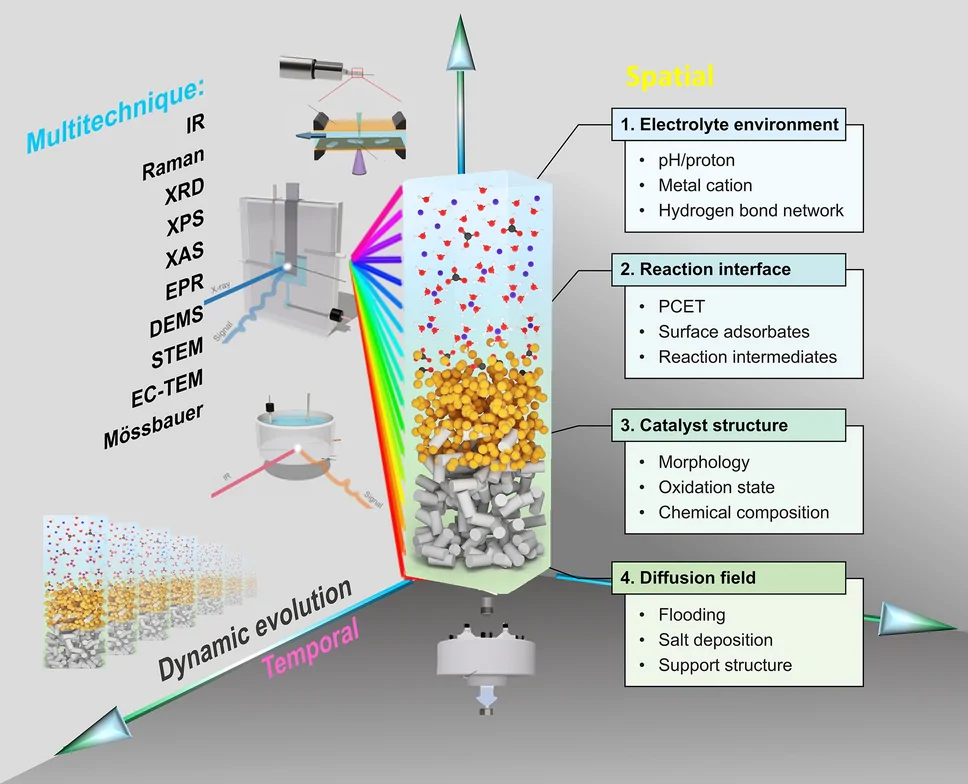
The electrochemical CO_2_RR research framework constructed near the three-phase interface, covering spatial (microregion), temporal (dynamic evolution), and multitechnique (combined characterization) dimensions

CO_2_RR is a typical surface-mediated process in which the reaction kinetics are governed by the dynamic evolution of reactants at the electrocatalyst–catholyte interface and mass transfer phenomena at the resulting triple-phase interface, where gas, electrolyte, and catalyst phases meet. The complex reaction environment, coupled with multistep proton-coupled electron transfer (PCET) mechanisms, introduces numerous variables that influence product distribution. As shown in Scheme 1, we spatially divide the GDE employed in the CO_2_RR system into four functionally distinct microregions. And in the subsequent sections, we critically discuss the latest advancement achieved through multi‑technique approaches in observing the in situ dynamic evolution processes within each microregion.

1. Electrolyte microenvironment: This microregion encompasses either the catholyte (in flow cells) or the anion-exchange membrane (in membrane electrode assembly (MEA) reactors) and can be further subdivided into the electrical double layer (EDL) and diffusion layer (DL) based on their proximity to the reaction interface [27, 28]. The hydrated cations in the EDL can substantially affect the local electric field, hydrogen-bond network, and pH, while the DL involves issues related to ion transport and reactant/product diffusion [29, 30].
2. Reaction interface: This microregion includes the PCET-active zone where critical processes occur, such as CO_2_ adsorption and activation, the formation and migration of key intermediates, and product selectivity regulation through surface energetics. Although activating stable CO_2_ molecules is relatively challenging, the binding strength of the *CO intermediate and its subsequent conversion into hydrogenated species (*CHO, *OCCO, *OCCOH, etc.) via PCET determine the final products, including H_2_, CO, or other more reduced oxygenates and hydrocarbons [31].
3. Electrocatalyst structure: This microregion comprises the electrocatalyst, modifiers, and other components. The electrocatalyst composition determines the product distribution, with different metal-based materials yielding distinct product profiles. Among them, Cu-based materials have garnered considerable attention for their remarkable selectivity toward multicarbon (C_2+_) products [32, 33]. Additionally, modifiers can improve reaction performance by adjusting the local electric field, concentration gradients, and chemical nanoscale domains.
4. Diffusion field: The gas diffusion layer is designed to overcome the limitations imposed by the low solubility of CO_2_ in liquids. This microregion predominantly comprises microporous and macroporous layers that control the mass transfer pathways for water, reactants, and products into and out of the electrocatalyst layer. The macroporous layer permits the diffusion of gaseous CO_2_ from the reactant gas stream to the electrocatalyst surface, and vice versa for gaseous products. A microporous layer between the macroporous and electrocatalyst layers often serves as a hydrophobic barrier to prevent complete immersion of the electrocatalyst in electrolyte or water [34, 35]. It should be noted that the diffusion field is more macroscopic compared to other microregions, and it involves a more diverse range of research topics. However, considering its significant role as an important component of the triple-phase interface in CO_2_RR, we have included it in this research framework as well. It has helped CO_2_RR to better shift from mechanism research to potential industrial applications.

### Ion Effects and Microenvironment Modulation in Electrolyte

**Fig. 1:**
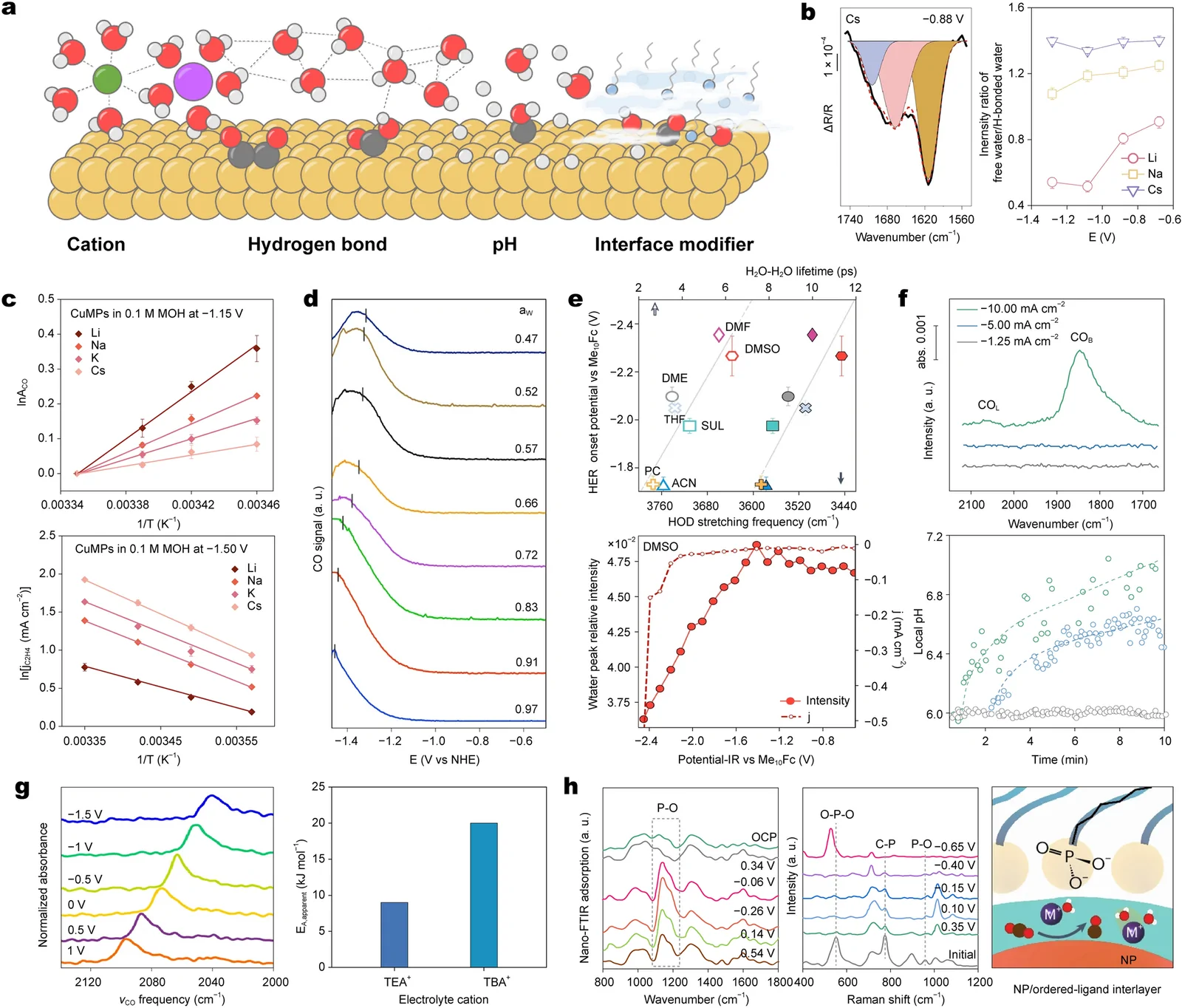
**a** Schematic of electrolyte species and typical effects in the electrical double layer. **b** ATR-SEIRAS of interfacial water on Cu film and potential-dependent intensity ratio of free water/H-bonded water. Adapted with permission [38]. Copyright 2024, Springer Nature. **c** Semi-log plots of normalized integrated CO band area and the partial current density of C_2_H_4_ versus 1/T, respectively. Adapted with permission [39]. Copyright 2024, Springer Nature. **d** CO signal (m/z = 28) as a function of potential via linear sweep voltammetry in the cathodic direction for various a_w_. Adapted with permission [42]. Copyright 2023, Springer Nature. **e** Onset potentials at 1 mA cm^−2^ versus HOD stretching frequencies and the H_2_O–H_2_O lifetime. Relative intensity values between the water and solvent/TBA peaks from the in situ SERS spectra and current density values at different potentials. Adapted with permission [43]. Copyright 2024, Springer Nature. **f** In situ ATR-SEIRAS of the adsorbed CO region and local pH as a function of time. Adapted with permission [44]. Copyright 2024, American Chemical Society. **g** In situ ATR-SEIRAS for CO adsorbed on Pt electrodes in acetonitrile containing 0.15 M tetraethylammonium perchlorate and the calculated apparent activation energy. Adapted with permission [45]. Copyright 2025, Springer Nature. **h** In situ nano-FTIR and Raman spectra from Ag electrode. Adapted with permission [46]. Copyright 2024, Springer Nature

The local environment of the reaction interface is considered to be a key factor influencing CO_2_RR activity and selectivity. Interfacial cations are considered to enter the EDL and substantially affect the local pH, water structure, and electric field of the microenvironment (Fig. 1a) [35, 36]. However, the mechanism underlying the cation effects that tune CO_2_RR kinetics remains controversial. With advancements in in situ characterization techniques, recent studies have expanded the understanding of cation and solvent effects [37, 38], proposing more strategies for microenvironmental regulation. Recent studies have revealed that different types of alkali metal cation affect the CO_2_RR performance by modulating the energies of both the reactant and activated complex in opposite directions. Using attenuated total reflection surface-enhanced infrared absorption spectroscopy (ATR-SEIRAS), Zhang et al. [38] revealed that Li^+^ enhances CO_2_ adsorption more effectively than other larger cations but slows down the hydrogenation kinetics of CO_2_ (Fig. 1b). This stems from the rigid water network surrounding Li^+^, which hinders the hydrogenation of water toward the adsorbed CO_2_. In contrast, the more flexible water network around larger cations (e.g., Cs⁺) facilitates water rearrangement and enhances hydrogen access to CO_2_. To further elucidate the multiple effects of different cations in enhancing adsorption and promoting hydrogenation conversion, Xu et al. recently uncovered a new enthalpy-entropy compensation phenomenon (Fig. 1c) [39, 40]. The results indicate that in the presence of cations, the adsorption strength of CO_2_ becomes increasingly unfavorable with larger ionic radii, following the order Li⁺ > Na⁺ > K⁺ > Cs⁺, while the stability of the transition state in the CO_2_RR hydrogenation conversion shows the opposite trend. It is evident that the effectiveness of cation effects highly depends on reaction conditions, including the applied potential, electrolyte concentration, and electrode properties. They may each play distinct roles in enhancing adsorption, improving activity, promoting C–C coupling, and regulating the selectivity toward C_2+_ products. Recent evidence suggests that, in certain scenarios, the trade-off among these different enhancement pathways is difficult to break [41]. Therefore, for multicarbon product pathways involving complex PCET steps, the design and optimization of electrolyte engineering can no longer be achieved by merely one or two inorganic metal ions. The design of multifunctional organic electrolyte systems may offer a tailored interfacial reaction environment specifically for CO_2_RR.

In addition to alkali metal cations, electrolyte anions can also influence CO_2_ electroreduction, albeit primarily through indirect and condition-dependent pathways [47]. Unlike cations, which strongly modulate interfacial electric fields, water structure, and PCET kinetics, most commonly used anions do not directly participate in CO_2_ activation on Cu under typical operating conditions. Instead, their effects are manifested through modulation of ion pairing, solvation structure, buffer capacity, and local pH near the electrode surface [47, 48]. In some cases, specifically adsorbing anions may compete with reaction intermediates for surface sites or alter the interfacial potential distribution, thereby influencing reaction selectivity. For example, hydrophobic anions with a strong affinity for CO_2_ have been reported to enhance CO_2_ mass transport toward the interface, thereby improving CO_2_RR selectivity by suppressing competitive hydrogen evolution reaction (HER) [49]. Other anions that undergo specific adsorption (e.g., halides) can alter the binding strength of key intermediates and restructure the electric double layer, although excessive adsorption may reduce the number of available active sites [47, 50]. Additionally, non-buffering anions have been shown to elevate local pH by suppressing buffering equilibria, thereby affecting the balance between CO_2_RR and HER at the electrode surface [51]. In the electrolyte systems emphasized above, non-specifically adsorbing anions are employed to minimize direct surface interactions. Under these conditions, anion effects are generally secondary compared to those of cations and mainly act as a background modifier of the interfacial environment rather than a primary determinant of CO_2_RR kinetics or product distribution [52].

When altering the microenvironment of the reaction interface, changes in water structure must be considered. Ideally, water acts as a proton donor, participating in CO_2_RR while avoiding involvement in the undesired HER. Notably, recent investigation utilizing in situ shell-isolated nanoparticle-enhanced Raman spectroscopy (SHINERS) has elucidated the correlation between interfacial water structure and reaction barriers [53]. The spectroscopic results highlighted that disrupted hydrogen-bonding networks create a disordered water environment. A high population of free water, as indicated by the enhanced high-wavenumber O–H stretching component, reflects a disrupted hydrogen-bond network with increased configurational entropy. Such disorder increases the reorganization energy associated with water dissociation and kinetically disfavors HER. In contrast, key PCET steps in CO_2_ electroreduction—such as the hydrogenation of adsorbed *CO intermediates—require proton donors that can flexibly reorient and interact with specific surface-bound species [53]. To clarify the spectral interpretation, “free water” (also referred to as weakly or non-hydrogen-bonded water) is defined here as water molecules experiencing minimal hydrogen-bonding interactions with neighboring species. Spectroscopically, this population is identified by a high-wavenumber O–H stretching feature centered at approximately ~ 3600 cm^−1^, which is distinct from the bands associated with hydrogen-bonded water clusters (~ 3420 cm^−1^) and extended hydrogen-bond networks (~ 3270 cm^−1^) [53, 54]. The relative abundance of free water can be quantified by performing deconvolution in the O–H vibrational region and calculating the integral-area ratio of the high-wavenumber components to the total O–H integral. Higher salt concentrations disrupt hydrogen bonds and reduce the water activity (*a*_*w*_), which suppresses HER and enhances selectivity for C_2+_ products [42, 55]. Thermodynamically, *a*_*w*_ at low pressures is defined as the ratio of the equilibrium partial vapor pressure of water in the electrolyte (*p*) to that of pure water (*p*_0_) at the same temperature (*a*_*w*_ = *p*/*p*_0_). It serves as a measure of the chemical potential of water species. It is crucial to distinguish *a*_*w*_ from molar concentration or ionic strength; while increasing the solute concentration generally lowers *a*_*w*_ due to hydration effects, *a*_*w*_ specifically quantifies the “free” energy state of water molecules available for surface adsorption and proton transfer. Experimentally, the *a*_*w*_ values cited in this context are typically determined via vapor pressure osmometry or calculated using semi–empirical models based on osmotic coefficients [56]. Time-resolved electrochemical mass spectrometry demonstrated a potential dependence of CO adsorption kinetics on *a*_*w*_ (Fig. 1d), indicating that low a_w_ values promote C–C coupling by increasing the surface coverage of *CO. Compared to aqueous electrolyte systems, non-protonic media with high CO_2_ solubility, low HER activity, and absence of pH equilibrium constraints present a promising environment for CO_2_RR. In situ surface-enhanced Raman spectroscopy and infrared spectroscopy (IR) revealed that the solvation behavior of water in non-protonic solvents regulates HER activity and CO_2_RR selectivity. Solvents with high-donor numbers can extend the onset potential for HER by confining water within a strong hydrogen-bond network [43]. A comparison of the behavior of cathodic currents and relative intensity of water in different solvents (Fig. 1e) demonstrates that using high-donor solvents to suppress proton reduction in acidic nonaqueous environments can also regulate CO_2_RR selectivity.

Previous studies suggested that CO_2_RR in acidic systems was dependent on the presence of high cation concentrations [57, 58]. However, recent observations indicate that the essential condition for CO_2_RR is an interfacial alkaline microenvironment [59, 60], suggesting that the role of cations in facilitating CO_2_RR initiation should be revisited. Local proton depletion is necessary to trigger CO_2_RR in acidic electrolyte, which can be achieved at sufficiently high current densities, even in the absence of metal cations (Fig. 1f) [44]. This finding suggests that the enhancement of CO_2_RR performance by metal cations is more likely due to the suppression of H^+^ mass transfer and alteration of the interfacial solvation structure than to an interfacial electric field effect. From the perspective of stability, the metal salt concentration represents a harmful local pH management strategy given that carbonate formation consumes local CO_2_. On this basis, in situ electrode position using organic additives was shown to achieve highly selective CO_2_RR under acidic conditions by modifying electrodes with thin films and using a low metal salt concentration. This conclusion was recently corroborated using in situ confocal Raman spectroscopy [61]. Probing the active site distribution at the reaction interface of the GDE confirmed that CO_2_RR only occurs in an alkaline microenvironment where local H^+^ has been sufficiently depleted.

Owing to their high tunability, organic cations provide abundant research perspectives in the study of non-protonic electrolytes. Electrolyte cations with shorter alkyl chains are capable of enhancing the interfacial electric field strength, thereby accelerating the CO formation rate (Fig. 1g) [45]. This modulation of interfacial field strength is attributed to changes in the distance between cations and the electrode. The electric field changes caused by ligand dissociation at the catalytic interface can induce favorable noncovalent interactions around active sites, customizing the microenvironment to guide specific reaction pathways. The continuously induced bond cleavage of surface ligands under bias was captured using in situ Fourier-transform infrared nano-spectroscopy (nano-FTIR) and surface-enhanced Raman spectroscopy (SERS) [46]. The transition process from bidentate to monodentate adsorption on the catalytic interface ultimately leads to the formation of free ligands, which involves conformational changes in alkyl chains (Fig. 1h). Complete ligand detachment provides space for cation insertion, and the resulting negatively charged layers promote shedding of the cation solvation shell, enhancing electron transfer in electrocatalytic systems.

It is noteworthy that, while traditional aqueous cells use bulk liquid electrolytes, MEA systems shift the electrolyte’s role to a solid-state or thin-film ionomer phase (e.g., *Nafion* or *Sustainion*). In MEA configurations, although there is no direct liquid contact, the catalyst is significantly influenced by the ionomer-mediated microenvironment through several key mechanisms [62, 63]. The ionomer embedded in the catalyst layer facilitates the EDL formation at the solid–solid interface. Cations originating from the membrane or anolyte crossover reside within this confined EDL, where they modulate the local electric field and the interfacial hydrogen-bond network [64]. Furthermore, the ionomer functions as both a local reservoir and a conductor for water molecules supplied through gas humidification or membrane crossover. This local water governs the kinetics of PCET, local pH, and electrolyte migration.

### Catalytic Mechanisms and Key Intermediates at Reaction Interface

**Fig. 2:**
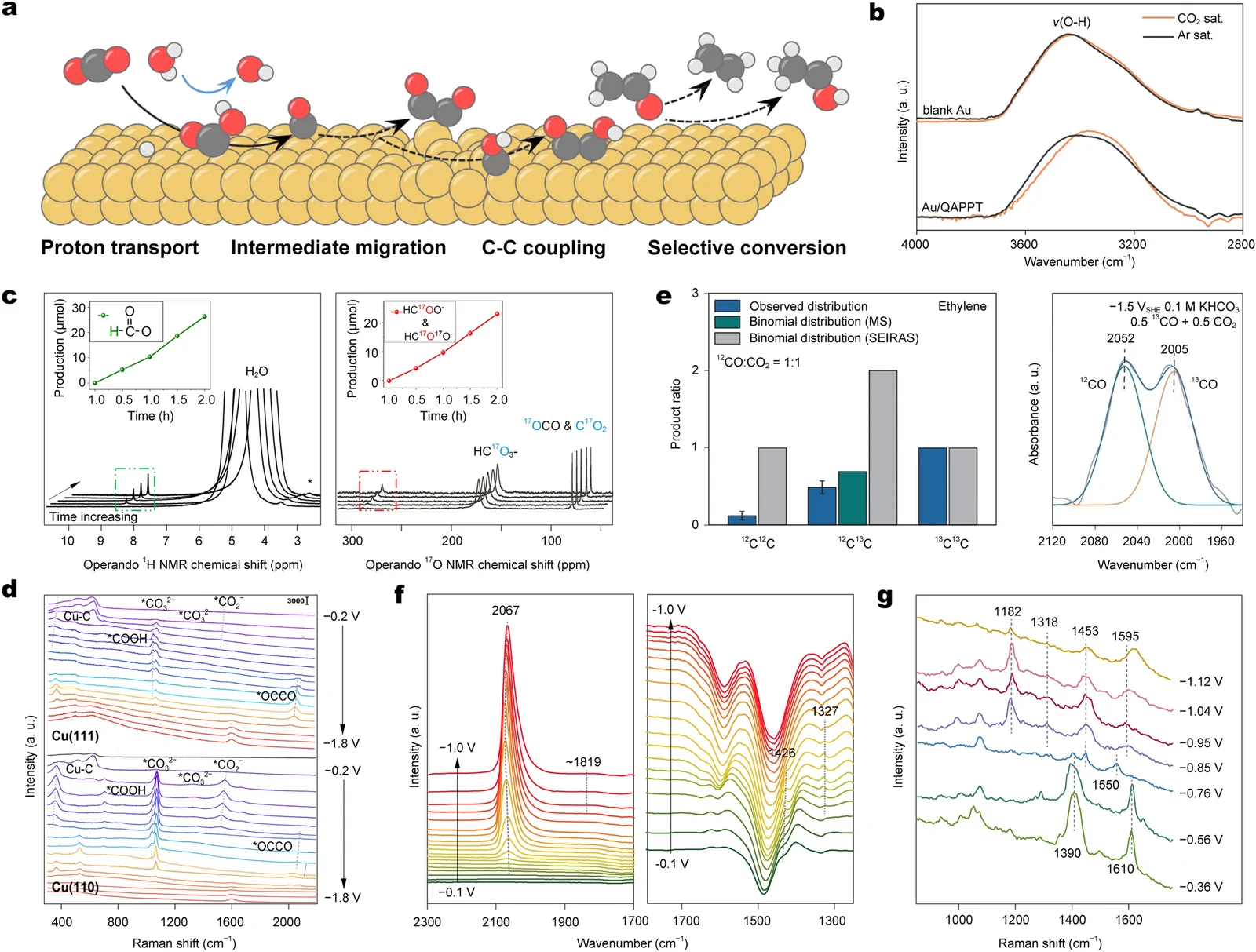
**a** Schematic of key intermediates and elementary reactions in CO_2_RR. **b** On the blank Au surface and Au/QAPPT interface, the *ν*(O–H) band upon switching from the Ar to the CO_2_ atmosphere. Adapted with permission [65]. Copyright 2024, American Chemical Society. **c** Operando NMR monitoring of oxygen participation in the Bi_2_CuO_4_ catalyst system: ^1^H NMR and ^17^O NMR spectra. Adapted with permission [66]. Copyright 2024, Elsevier Ltd. **d** In situ Raman spectra of Cu(111) and Cu(110) in 0.5 M KHCO_3_/H_2_O saturated with CO_2_. Adapted with permission [67]. Copyright 2022, Royal Society of Chemistry. **e** Observed distribution and expected binomial distributions of isotopologues of ethylene produced based on the ^12^CH_2_^12^CH_2_/^13^CH_2_^13^CH_2_ ratio and ^12^CO_L_/^13^CO_L_ ratio and in situ SEIRA spectra of adsorbed CO on dendritic Cu. Adapted with permission [68]. Copyright 2023, Springer Nature. **f** In situ SEIRAS spectra of the anodized copper thin film in a CO_2_-saturated, 0.1 M KHCO_3_ electrolyte in H_2_O. Adapted with permission [69]. Copyright 2024, American Chemical Society. **g** Raman spectra of an electrochemically treated Cu foil acquired during CO_2_RR in a CO_2_-saturated 0.1 M NaClO_4_ electrolyte. Adapted with permission [70]. Copyright 2024, Springer Nature

The complexity of the CO_2_RR process lies not only in multistep PCET but also in the hydrogen-bond network, dynamic evolution of intermediates, and presence of heterogeneous active sites (Fig. 2a). In situ electrochemical spectroscopy enables real-time detection of reactant species and the evolution of key intermediates during the reaction, providing theoretical insights and design guidelines for the CO_2_RR process and electrocatalyst design.

Water serves as the proton source for CO_2_RR. However, the proton activity of interfacial H_2_O species is influenced by cations, leading to differentiated proton behavior [71]. A recent study [65] demonstrated that coating large organic cationic groups on Au electrodes can create a MEA-like Au/quaternary ammonium polymer (QAPPT) reaction interface (Fig. 2b). CO_2_RR at the interface of blank Au and Au/QAPPT polymer electrolyte was monitored using ATR-SEIRAS. Cation distribution was shown to be prevented at the Au/QAPPT interface, which possessed anion-exchange membrane properties, and the v(O–H) band shifted to lower wavenumbers in the presence of CO_2_. This shifting reflected disruption of the hydrogen-bond network and enhanced proton transfer, leading to increased formation of *COOH. In contrast to hydrogen in water, monitoring whether oxygen in water undergoes reversible conversion with oxygenated intermediates at the reaction interface and its impact on key intermediates is more challenging. The origin of oxygen atoms in the CO_2_RR process was traced using in situ electrochemical nuclear magnetic resonance spectroscopy (Fig. 2c) [66], revealing that *COOH and *HCOO generation through oxygen exchange effectively inhibits *CO dissociation and hydrogenation, promoting HCOO^−^ formation. This finding implies that the oxygen atoms of adsorbed H_2_O molecules can directly participate in HCOO^−^ formation though a water-assisted mechanism.

Single-crystal electrodes combined with isotope probes have become a key method for understanding the relationship between catalytic sites and reaction activity. Two consecutive orthogonal processes are generally believed to exist on the Cu surface: CO_2_ → *CO and *CO → C_2+_. Different crystal planes significantly influence the selectivity of catalytic reactions due to variations in coordination numbers and surface energies. In situ Raman spectroscopy reveals facet-specific intermediate adsorption. Specifically, on the low-coordinated Cu(110) surface, distinct signals of *OCCO are observed, which are absent on the close-packed Cu(111) facet. By contrast, the Cu(111) plane is more favorable for generating *COOH and *CO, thus dominating the production of C_1_ products. This highlights how lower coordination numbers on high-index or open surfaces effectively stabilize C–C coupling intermediates, thereby directing selectivity toward C_2+_ products (Fig. 2d) [67, 72]. In situ ATR-SEIRAS revealed the fundamental principles for building favorable active sites during CO_2_/CO coelectrolysis (Fig. 2e) [68, 73]. Although the Cu(111) plane contains a higher proportion of CO_2_ adsorption and activation sites, *CO must migrate to highly distorted defect sites (e.g., steps, vacancies, and kinks) on Cu surfaces to effectively undergo C–C coupling. This dual-site model provides a foundation for the design of tandem electrocatalysts [74, 75].

Although C–C coupling is essential for the formation of C_2+_ products, identifying short-lived, low-coverage coupling intermediates remains challenging. Using ATR-SEIRAS, Delmo et al. [69] analyzed the evolution of key CO_2_RR intermediates on pre-electrochemically oxidized Cu films (Fig. 2f), demonstrating that the oxide-derived Cu electrode enhanced the initial chemisorption of *COO^−^, leading to a rapid increase in *CO coverage. Density functional theory (DFT) calculations and frequency simulations identified a distinct *COCHO signal peak, suggesting a new C–C coupling pathway. *CO hydrogenation was found to be the RDS for C_2+_ formation, with the coupling of *CO and *CHO thermodynamically favoring *COCHO formation. This finding challenges the viewpoint that *CO dimerization involving a single electron transfer is the RDS for C_2+_ molecule conversion.

*OCHCH_2_ is considered to be a critical intermediate shared in the conversion pathways of ethylene and ethanol [76, 77], with the bonding strength between Cu, C, and O playing a key role in controlling the presence or absence of OH groups in C_2+_ products. A recent study [70] combining in situ Raman spectroscopy with DFT-optimized vibrational fingerprints of intermediates found that ethylene, the only C_2+_ product at low overpotential, could be formed via the *OCCO(H) intermediate. Undercoordinated distorted sites with high surface strain and deep *s*-band states exhibited *OCHCH_2_ conversion activity at higher overpotential (Fig. 2g). *OCHCH_2_, which serves as a critical intermediate for the formation of C_2+_ alcohols, can be reduced to acetaldehyde and ethanol or can couple with *CO/*COH species to form allyl alcohol, which leads to 1–propanol formation in subsequent reduction steps.

To reduce uncertainty in intermediate identification, we establish a robust correlation between spectroscopic fingerprints and specific reaction pathways (Table 1). A critical challenge in CO_2_RR is discriminating between C–C coupling precursors. Our analysis reveals that SERS and ATR-SEIRAS provide complementary selection rules: The neutral dimerization of *CO to *OCCO is typically characterized by Raman-active Cu–C vibrations (~ 360 cm^−1^), while the formation of *OCCOH is indicated in SEIRAS by the C–OH bending mode (~ 1180 cm^−1^) and a redshifted C=O stretch (~ 1651 cm^−1^) [78]. The latter is definitively confirmed via D_2_O isotopic substitution, which induces a significant frequency shift, whereas the neutral dimerization pathway remains invariant. Furthermore, spectral interpretation must account for potential artifacts [78]. In ATR-SEIRAS, line shapes often observed in the water-bending region (1600–1700 cm^−1^) are frequently misassigned to carbonyl intermediates but are typically induced by local pH-dependent changes in the electrolyte’s refractive index. Similarly, in Raman spectroscopy, background signals from amorphous carbon must be distinguished from the CH_*x*_ vibrational bands (~ 2800–3000 cm^−1^) of actual reaction intermediates. Assignment of in situ ATR-SEIRAS and Raman diagnostic bands for surface species and intermediates during the CO_2_RR have been summarized in Tables 2 and 3.

**Table 1:** Concise mapping of diagnostic spectroscopic features to CO_2_RR pathways

Intermediate	Technique	Diagnostic feature (cm^−1^)	Isotopic shift (^13^C/D)	Selection rules and artifacts	Pathway role
*OCCO	SERS	~ 360 (Cu–C), ~ 1550 (C=O)	~ 40–50 (^13^C)	Raman active; insensitive to IR-silent symmetric modes	Neutral dimerization (C_2+_ path)
*OCCOH	SEIRAS	~ 1180 (C–OH), ~ 1670 (C=O)	Significant D shift	Dipole/perp to surface required; sensitive to PCET	Protonated coupling (ethylene path)
*CHO	SEIRAS	2800–2900 (C–H), 1031	~ 10–15 (^13^C)	Overlaps with electrolyte CH_*x*_ contaminants	C_1_ pathway (methane)
*CO_2_^−^	SEIRAS	1300–1330 (sym), 1550 (asy)	~ 30–40 (^13^C)	Overlaps with bulk CO_3_^2−^/HCO_3_^−^ signals	Initial activation
*OCH_2_	SEIRAS	1380–1450 (δCH_2_)	Significant D shift	Often a late-stage intermediate for alcohols	Deep reduction

**Table 2:** Assignment of in situ Raman diagnostic bands for surface species and intermediates during the CO_2_RR

Category	Species	Band Position (cm^−1^)	Vibrational Mode Assignment	References
Catalyst phases	Cu_2_O	142–150, 216–223, 525, 615–630	Lattice vibrations/multiple characteristic modes	[79–82]
	CuO	288–295, 330–342, 621–628	Characteristic lattice vibrations	[83, 84]
	Cu(OH)_2_	292, 483–488	Hydroxide-related features	[80, 85]
Surface states	Cu–CO_(fr)_	276–290	Frustrated rotation of Cu–CO	[86, 87]
	Cu–CO_(st)_	354–370	Cu–CO stretching vibration	[88]
	Cu–O_(ad)_	601–624	Adsorbed surface oxygen species	[89]
	Cu–OH_(ad)_	706	Adsorbed surface hydroxyl species	[79, 90]
Key intermediates	*CO_(bridge)_	1900–2000	C–O stretching of bridge-bonded *CO	[91, 92]
	*CO_(atop)_	2000–2120	C–O stretching of atop-bonded *CO	[93–95]
	*CO_2_^−^	1300–1330, 1520–1590	Symmetric/antisymmetric stretching	[96, 97]
Environment	*OCCHO	2700	C–C coupling related intermediate	[98]
	CH_*x*_	2800–3000	C–H stretching of hydrocarbon species	[87, 99]
	*CO_3_^2−^	1060–1080, 1350–1430	Interfacial carbonate species	[82, 96]
	*HCO_3_^−^	~ 1345	Interfacial bicarbonate species	[88, 93]
	H_2_O	1600–1690	Bending vibration of solvent water	[93, 99]

**Table 3:** Assignment of in situ IR diagnostic bands for key intermediates on diverse electrocatalysts during CO_2_RR

Intermediate/species	Band position (cm^−1^)	Vibrational mode assignment	Catalyst system	Electrolyte/conditions	References
*COOH	1253, 1288	OH deformation/C–O stretch	Ag film, PcCu–Cu–O MOF	0.1 M KCl/CO_2_	[100, 101]
*CO (atop/linear)	1386, 1396	C–O stretch (Symmetric)	Ag film, PcCu–Cu–O MOF	0.1 M KCl/CO_2_	
	1648, 1660, 1713	C=O stretching vibration	Pt, Ag, PcCu–Cu–O MOF	KHCO_3_/KOH-KHCO_3_	[102]
	2102, 2105, 2112	C≡O stretching (Linear *CO)	Au(111), Ag@Cu, Pt	KHCO_3_/KOH-KHCO_3_	[103]
	2033–2050	Metal–CO/Atop-bonded *CO	Ag, Cu film/NWs	0.1 M KHCO_3_	[104, 105]
	1926	Linear-bonded *CO	Pd@Pd3Au7 nanocube	0.1 M KHCO_3_	[106]
*CO (bridge)	1831, 1832	Bridge-adsorbed *CO	Pt(111), Cu thin film	0.1 M KHCO_3_	[104]
	1864, 1950	Bridge-bonded *CO	Pd@Pd3Au7, Cu NWs	0.1 M KHCO_3_	[104, 105]
C_2+_ and specialized	1575	Asymmetric vib. of *COCHO	PcCu–Cu–O MOF	–	[101]
Inorganic ions	1031	Nonplanar vib. of *CHO	PcCu–Cu–O MOF	–	
	894, 3108	C–H bend/stretch of *CH_2_=	PcCu–Cu–O MOF	–	
	1355	CO stretching of HCO_3_^−^	Pt(111)	KOH-KHCO_3_	[102]
	1520	Adsorbed CO_3_^2−^}	OD-Cu nanocrystals	0.1 M KHCO_3_	[106]
Solvent/water	1620, 1641, 1650	H–O–H bending (adsorbed H_2_O)	Ag, Cu, Pd–Au	Various	[100, 102, 106]
	3000–3600	O–H stretch (adsorbed H_2_O)	Au film	0.1 M KHCO_3_	[103]

### Dynamic Evolution of Electrocatalyst Structure and Oxidation States

**Fig. 3:**
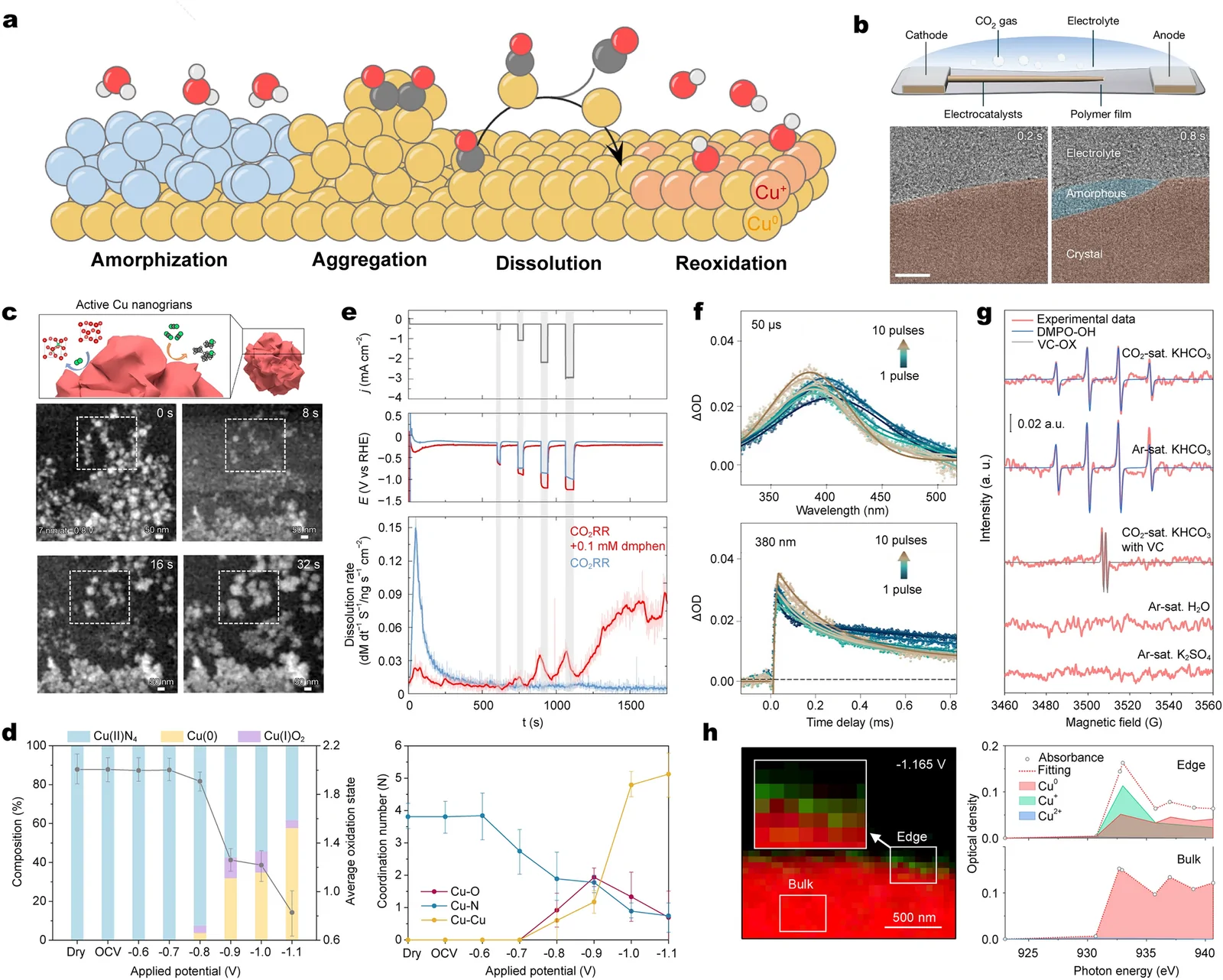
**a** Schematic of morphological features in catalytic structural evolution. **b** Schematic of a perspective view of the sample area and TEM images show the emergence of the amorphous interphase. Adapted with permission [111]. Copyright 2024, Springer Nature. **c** Schematic of a highly polycrystalline Cu nanograins. Further particle aggregation of 7 nm NPs. Adapted with permission [112]. Copyright 2023, Springer Nature. **d** Extracted oxidation state of N–Cu SAC and quantitative analysis of coordination environment at various potentials was extracted based on the fitting of X-ray absorption near-edge structure (XANES) and extended X-ray absorption fine structure (EXAFS). Adapted with permission [114]. Copyright 2023, Springer Nature. **e** Online ICP-MS measuring the dissolution rate of Cu NPs under cathodic bias. Adapted with permission [115]. Copyright 2024, Springer Nature. **f** Transient absorption spectra as a function of the number of the electron pulse in the presence of 0.5 mM Cu(II) NPs at 50 μs and transient kinetics as a function of the number of electron pulses at 380 nm. Adapted with permission [116]. Copyright 2024, American Chemical Society. **g** EPR spectra of the corresponding solutions containing 100 mM DMPO after 24 h resting. The concentrations of KHCO_3_, K_2_SO_4_, and VC are 0.5 M, 0.25 M, and 10 mM, respectively. Adapted with permission [117]. Copyright 2022, Springer Nature. **h** Chemical composition mapping image of the Cu film at a cathodic potential and optical density of the edge and bulk regions on the colored image. Adapted with permission [118]. Copyright 2024, Elsevier Ltd

The physicochemical properties of Cu-based electrocatalysts (e.g., crystal phase, coordination environment, and oxidation state) critically govern CO_2_RR performance, particularly C_2+_ product selectivity (Fig. 3a) [107, 108]. Cu-based electrocatalysts show promising potential for C_2+_ product applications. However, the dynamic triple-phase interface poses morphological challenges, including low Ostwald ripening resistance, structural instability under operating conditions [109], and ambiguous oxidation state identification [110]. In situ high-resolution transmission electron microscopy imaging revealed a metastable amorphous interfacial layer at the charged solid–liquid boundary (Fig. 3b) [111], which exhibited dynamic fluidic behavior and amorphous–crystalline phase transitions. This electron doping-induced surface amorphization enhanced C_2_H_4_ selectivity, with layer thickness positively correlating with ethylene yield. A recent study [112] captured the full lifecycle of 7 nm Cu nanoparticles during CO_2_RR redox cycling (Fig. 3c), demonstrating potential-driven nanoparticle migration/coalescence that generated grain boundaries and undercoordinated sites, which were proposed as active centers for C_2+_ formation. While such dynamic interfaces enhance catalytic activity, they simultaneously promote material aggregation and nucleation, representing a key degradation pathway in single-atom catalytic systems [113].

Concerns about the stability of common M–N_4_–C electrocatalysts arise due to aggregation induced by the dissociation of M–N motifs [62, 63]. In situ XANES and EXAFS analysis [114] of the Cu–N_4_ electrocatalyst revealed that single-atom Cu–N coordination underwent partial reversible reconstruction with potential dependence (Fig. 3d), whereas the formation of the Cu–Cu bond was irreversible. An increase in Cu–Cu coordination number led to the loss of catalytic activity. A recent ATR-SEIRAS study [119] suggested that this reconstruction may be linked to H• generation under CO_2_RR operating conditions. Highly active H• attacks the Cu–N bond at single-atom catalytic sites, causing Cu dissolution. The dissolved Cu^2+^ is subsequently reduced to Cu nanoparticles under cathodic potential. Moreover, Cu single atoms coordinated with pyridine N exhibited substantially better antiaggregation performance compared to those coordinated with pyrrole N due to the stronger Cu–N_pyrr_ bond, providing new insights for stabilizing M–N motifs through local coordination modulation [120, 121].

The detachment and/or dissolution of nanoparticles into metal ions under CO_2_RR operating conditions are potential causes of electrocatalyst deactivation and stability loss. Online mass spectrometry tracking Cu dissolution and redeposition under pulsed current conditions revealed that the dissolution rate was correlated with CO_2_RR current density (Fig. 3e) [115]. OH, CO, and oxalate intermediates form free-state complexes with Cu^+^, which weakens the stability of the Cu–Cu bond. Among these, the high concentration of adsorbed *CO was identified as a key species driving Cu reconstruction. Another study [64] suggested that this degradation occurs during CO_2_RR and HER processes on Cu and Au electrodes, indicating that CO is not the sole cause of dissolution. These authors found that Cu nanoparticle detachment appeared to be more dependent on the electrolyte environment than on current density. This is because OH^−^ produced during CO_2_RR and HER increases the local pH at the electrode interface, leading to the formation of salts (e.g., CuCO_3_ or Cu(OH)_2_) in highly alkaline environments. Although these salts are insoluble in water, they may be stripped away with the initial bubble nucleation of gaseous products. The acidic electrolysis system effectively inhibits the increase in local pH and also alleviates the precipitation of carbonates. However, this imposes higher requirements on the corrosion resistance of the catalytic structure, especially during open-circuit operation or during shutdown periods.

The dynamic presence of oxidized Cu species during CO_2_RR remains controversial, as the operational window lies far below the redox potential of Cu^0^/Cu^+^ [122]. As shown in Fig. 3f, time-resolved electron pulse irradiation experiments by Jiang et al. [116] revealed the rapid degradation of Cu^2+^ to Cu^+^ during CO_2_^•−^ interfacial reactions. While subsurface CuO is favorable for CO_2_ adsorption, C–C coupling predominantly occurs at Cu^+^ sites. Complementary work demonstrated OH-mediated surface Cu oxidation at high cathodic overpotential (Fig. 3g) [117, 123]: OH• generated via HCO_3_^−^/H_2_O oxygen exchange spontaneously oxidized Cu electrodes. This redox equilibrium existed dynamically and competitively on Cu, ultimately determining the Cu^+^ concentration during CO_2_RR. Similarly, Kim et al. [118] observed evidence of surface redox processes using operando soft X-ray microscopy (Fig. 3h), proposing that surface Cu^2+^ species could promote CO dimerization of adsorbed *CO and help to increase the local *CO/CO_2_ concentration near the Cu^0^ surface, enhancing C–C coupling activity. It is worth noting that although the valence states of the highly active Cu sites identified vary, a growing body of research has begun to discover and emphasize the role of oxidized Cu species in enhancing the performance of the CO_2_ reduction reaction. In the future, given that most of these observations are based on short-duration in situ characterizations, further confirmation is needed to determine whether Cu^+^/Cu^2+^ species are short-lived products in the dynamic evolution of the catalytic structure or can exist in a long-term stable state. Moreover, since many early studies did not involve the presence of oxidized Cu yet still demonstrated good CO_2_RR performance, the necessity of Cu^+^ presence warrants further investigation. Discussing Cu^+^/Cu^2+^ in general terms, without precisely defining their roles, could compromise the accuracy of our understanding of structure–activity relationships.

While Cu is unique in the electrosynthesis of C_2+_ products, emerging non-Cu catalysts demonstrate superior selectivity for C_1_ products by navigating distinct thermodynamic pathways. For example, single-atom catalysts (SACs) based on Ni and Fe have redefined the efficiency of CO production. Unlike their bulk counterparts, which are often poisoned by irreversibly bound *CO, Ni–N_*x*_ and Fe–N_*x*_ sites promote a high-spin to low-spin transition that facilitates *CO desorption, achieving FE exceeding 95% at high current densities [124]. In contrast, *p*-block metals such as Sn, Bi, and In selectively promote the production of formate [125, 126]. The reaction mechanism on these surfaces typically involves an oxygen-coordinated *OCHO rather than the carbon-coordinated *COOH observed on noble metals [127]. In situ ATR-SEIRAS studies on Bi-based nanosheets have confirmed that the stabilization of the *OCHO intermediate is highly sensitive to the local cation concentration a characteristic shared with Cu systems but resulting in a different terminal product [128]. Furthermore, Zn-based catalysts occupy an intermediate position; although historically classified as CO-selective, recent advances in nanostructuring and alloying have demonstrated their potential to modulate the *CO binding energy, achieving activity comparable to that of Ag at a fraction of the cost. By comparing these divergent pathways, it becomes clear that while Cu excels in C–C coupling, non-Cu systems offer more predictable tunability for specific C_1_ chemicals [129, 130].

Currently, catalytic materials capable of operating stably for hundreds of hours at current densities of 200–500 mA cm^−2^ in the electrosynthesis of C_2+_ and C_1_ products via the CO_2_RR are continuously being developed. However, the transition from laboratory-scale discovery to industrial application requires that catalysts maintain their performance for at least several thousand hours (≥ 1000h @ ≥ 500 mA cm^−2^) [7]. Only a few studies meeting this criterion have been reported, which is closely related to the still-unclear nature of stability decay. In the future, advances in operando characterization techniques may provide microscopic insights to optimize deactivation-resistant catalytic systems.

### Dynamic Monitoring of Species Transport in Diffusion Field

**Fig. 4:**
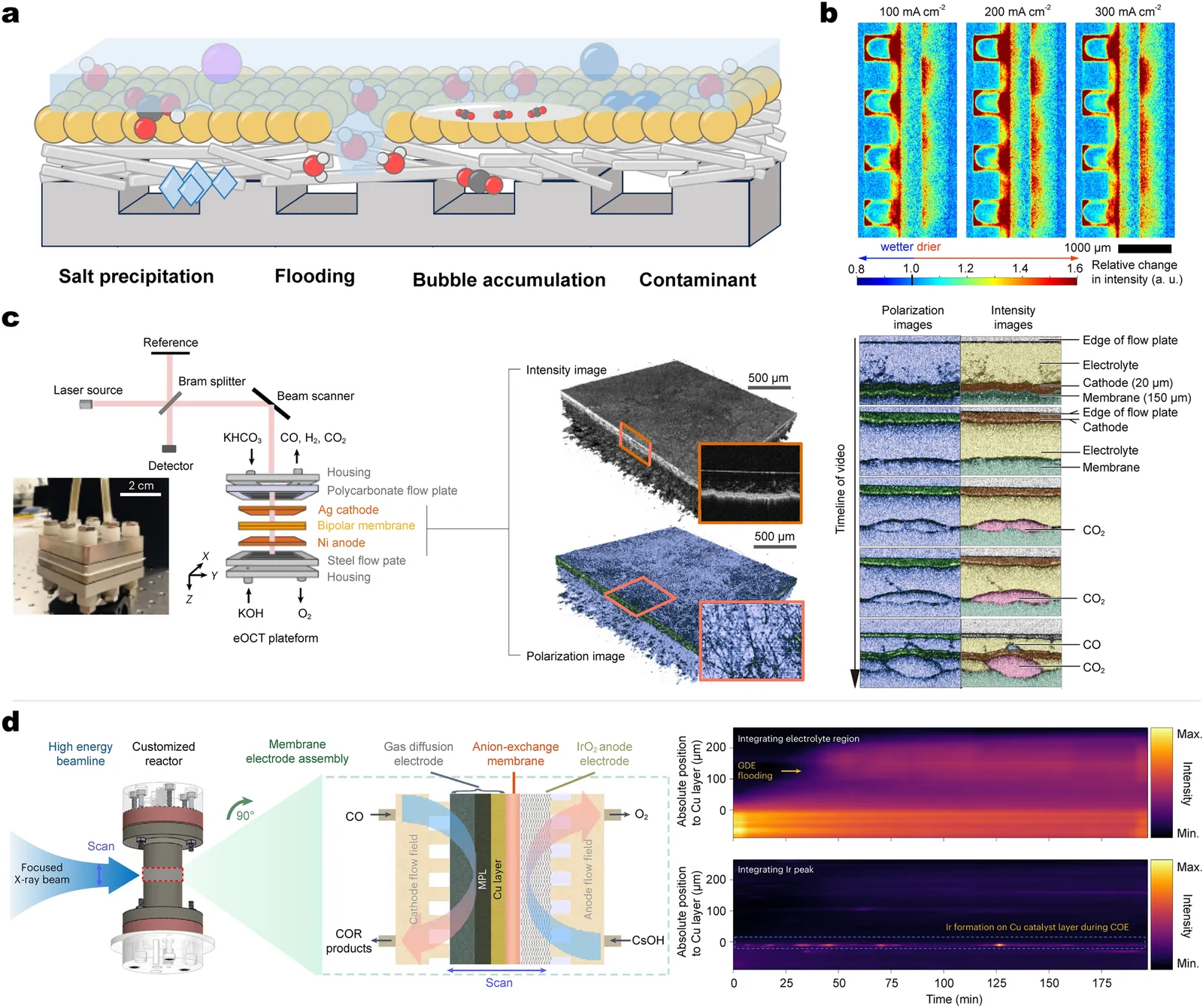
**a** Schematic of typical behaviors and scenarios near the GDE. **b** Neutron radiography of the CO_2_ electrolysis cell. The relative change in intensity normalized to the intensity of the zero current cell corresponds to a change in water content. Adapted with permission [134]. Copyright 2022, Springer Nature. **c** Electrolysis optical coherence tomography platform used to image CO_2_ electrolysis in operando in 3D as a function of time. The timeline is of a small region within the electrolyzer chosen to depict the location of CO_2_ and CO bubble formation. Adapted with permission [135]. Copyright 2024, Springer Nature. **d** Schematic diagram of the customized synchrotron cell and the MEA configuration for the operando COE measurement. The processed operando wide-angle X-ray scattering (WAXS) mappings by integrating corresponding electrolyte (that is, changes in background scattering) and face-centered cubic-structured Ir(111) peak. Adapted with permission [136]. Copyright 2023, Springer Nature

Device engineering is critical for the industrialization of electrochemical CO_2_RR [131]. Performance degradation due to phenomena such as flooding, salt precipitation, bubble accumulation, and contaminant poisoning limits the operational stability of electrolyzers (Fig. 4a). These processes often occur simultaneously at complex multiphase interfaces, and traditional electrochemical methods struggle to capture their spatiotemporal evolution. Recent advancements in in situ characterization techniques, particularly high-resolution neutron imaging, optical coherence tomography (OCT), X-ray scattering, and tomography, have provided powerful tools to observe dynamic changes within electrolyzers in real time, revealing key failure mechanisms and offering valuable insights for optimizing electrolyzer design and operation strategies. To clearly delineate the spatial distribution of processes within the GDE architecture, it is essential to distinguish between the diffusion layer–catalyst interface and the catalyst–electrolyte interface. Within our stereoscopic framework, the former is classified under the diffusion field as it primarily manages the logistics regulating the flux of gaseous CO_2_ and the expulsion of hydrophobic products [132, 133]. However, the chemical PCET is strictly localized at the reaction interface where the catalyst is in direct contact with the electrolyte. As CO_2_RR activity is negligible at a dry interface due to the absence of a proton source, the diffusion field’s primary role is to maintain a stable triple-phase boundary by preventing water flooding while ensuring a continuous CO_2_ supply to the reaction interface.

Flooding and salt precipitation are interrelated, complex issues in CO_2_ electrolysis. Cathodic reactions such as CO_2_RR and competitive HER consume water and generate OH^−^, which react with CO_2_ to form carbonate and bicarbonate ions. When ion concentrations exceed their solubility limit, salts such as KHCO_3_ and K_2_CO_3_ precipitate within the cathode gas diffusion layer (GDL) and electrocatalyst layer. Disch et al. [134] observed the blocking effect of salt precipitation on the cathode GDL using high-resolution neutron imaging (Fig. 4b). The fundamental mechanism behind this flooding was further elucidated by Burdyny et al. [137], who demonstrated that the hydrophobicity of carbon-based gas diffusion layers (GDLs) is dynamic. Under high polarization, the electrosorption of ions and changes in the surface energy of the carbon support can lead to capillary breakthrough, where liquid water penetrates the gas-transport pores. This transition from a gas-permeable state to a flooded state creates “water bridges” that physically isolate the catalyst from the CO_2_ feed. At a current density of 200 mA cm^−2^, salt crystals were found to penetrate the cathode GDL and accumulate primarily in flow field channels, obstructing CO_2_ transport and leading to a sustained decrease in Faradaic efficiency. Furthermore, their hygroscopic nature exacerbated water accumulation within the electrode, creating a positive feedback loop that eventually caused local or widespread flooding. Pulsed operation (periodically switching to high-potential resting periods) effectively mitigates salt precipitation and water flooding [138, 139] by interrupting electrochemical reactions and electroosmotic drag, allowing the cathode GDL to recover during rest periods. This process reduces local ion concentrations, promotes salt dissolution, and maintains electrode hydrophobicity through periodic wetting–drying cycles [140, 141]. Operating CO_2_RR in acidic electrolytes has attracted increasing attention because it fundamentally avoids carbonate/bicarbonate formation, which limits carbon utilization and contributes to salt precipitation in neutral/alkaline electrolytes. However, acidic media are inherently more conducive to the HER due to high proton availability, requiring microenvironmental engineering to balance CO_2_RR selectivity and stability.

Bubble accumulation presents another critical challenge in CO_2_ electrolysis, particularly in zero-gap electrolyzers, where the dynamic behavior of bubbles directly influences the mass transport interfacial structure. In a CO_2_-to-CO system, real-time 3D OCT imaging revealed that a dynamic separation of < 100 μm between the cathode and membrane triggered CO_2_ bubble nucleation on the membrane surface within 1s of CO_2_ electrolysis to produce CO (Fig. 4c) [135]. Large CO_2_ bubbles appeared to form after CO_2(g)_ necking and coalescence. OCT analysis demonstrated distinct spatial segregation, with CO_2_ bubbles localizing at the membrane–cathode interface, while the generated CO bubbles formed on the side of the cathode opposite the membrane, reflecting divergent formation pathways. CO_2_ arose from carbonate–proton recombination at the membrane interface, whereas CO was generated via electrochemical reduction on the electrocatalyst surface. CO_2_ and CO bubbles have been shown to cause mechanical damage to the membrane structure in bipolar membrane electrolyzers [142]. CO_2_ generated at the membrane interface diffuses toward the anode, while gaseous CO_2_ forms at the membrane–catalyst interface. Bubble accumulation induces localized pressure buildup, forming irreversible pinholes between the membrane and electrocatalyst layer that progress to membrane delamination under high current densities. Although such structural damage does not immediately degrade electrochemical performance, prolonged accumulation may lead to the risk of electrolyzer failure.

Contaminants and localized impedance jointly induce site deactivation and current inhomogeneity, severely impairing the operational efficiency and stability of electrolyzers. The dissolution of IrO_2_ anodes under oxidative conditions releases Ir ions, which traverses the anion-exchange membrane and deposits as metallic Ir on the cathode during reduction (Fig. 4d) [136]. This contamination demonstrates a linear correlation with HER selectivity enhancement. Although Ni-based anodes can temporarily alleviate the stability decline caused by poisoning, the continuous accumulation of acetic acid products will corrode the electrode and deplete the alkalinity of the anolyte. The use of alkaline-stable Ni-based anodes partially alleviates the durability challenges caused by poisoning. However, this is not a sustainable solution, given that acetate formation causes the pH of the anolyte to decrease, which corrodes the electrocatalyst anode. Superhydrophobic electrodes based on an expanded polytetrafluoroethylene (ePTFE) GDL offer a potential solution to mitigate flooding [143]. However, in situ IR revealed the dissolution of a thin (50 nm) Cu layer on ePTFE electrodes [144]. Notably, the nonconductive nature of PTFE means that the electrocatalyst layer is responsible for electron dispersion without additional current collectors, resulting in highly localized current densities at the edges—up to five times higher than the average. This phenomenon causes Cu electrocatalysts to oxidize and dissolve in the highly alkaline environment, forming Cu(OH)_2_ and shifting product selectivity toward methane and hydrogen in a current-dependent manner.

The diffusion field serves as a crucial link between the macroscopic electrolyte reservoir and the microscopic reaction interface, regulating the flux of reactants and products. At industrially relevant current densities (> 500 mA cm^−2^), the mass transport of CO_2_ becomes the rate-limiting step, often overshadowed by intense hydrogen evolution and the resulting localized concentration gradients. In situ investigations have revealed that the diffusion field is not a static transport layer but a dynamic environment susceptible to water flooding and salt precipitation. For example, the accumulation of liquid water within the carbon fiber headers of GDE obstructs CO_2_ pathways and leads to a significant decline in C_2+_ selectivity. Simultaneously, the enrichment of cations at the interface, while beneficial for CO_2_ activation, can exceed the solubility limit, causing KHCO_3_ or K_2_CO_3_ salt crystals to block the gas pores. Furthermore, the diffusion field governs the local microenvironment through a feedback loop. The thickness of the Nernst diffusion layer determines the magnitude of the pH gradient, with a thicker layer resulting in increased local alkalinity. This alkalinity, while suppressing HER, also promotes the parasitic reaction of CO_2_ with OH^−^ to form carbonates, effectively consuming the reactant before it reaches the active sites. By incorporating these macroscopic transport phenomena into our stereoscopic framework, we can more accurately predict the stability and efficiency of large-scale CO_2_RR systems.

## Summary and Outlook

This review provides a stereoscopic perspective on the different microregions in CO_2_RR systems based on recent advances in in situ characterization. In the electrolyte microenvironment, concentration polarization and local electric field effects are influenced by the combined actions of solvents and surface modifications on the cathode, which can affect CO_2_RR activity. CO_2_ activation and C–C coupling are currently considered to play crucial roles in interfacial reaction kinetics. However, there is no consensus regarding the dimerization precursors and key intermediates that determine product distribution in CO_2_RR systems. In terms of electrocatalyst structure, the impact of its dynamic evolution under operating conditions remains unclear due to the lack of high-resolution spatiotemporal detection techniques. Maintaining the active-phase structure and oxidation state while avoiding dissolution may help to break the “seesaw effect” between activity and stability. In the diffusion field, achieving stable electrolysis at high current densities is a prerequisite for the industrialization of CO_2_RR technology. However, flooding and salt precipitation are known but unsolved causes of performance degradation, and metal cation migration/deposition and local bubble formation due to gaseous product generation can cause irreversible damage to active sites and electrode structures. Although the development of in situ characterization techniques has greatly advanced our understanding of these microregions, the establishment of structure–activity relationships in CO_2_RR systems remains inadequate. We propose the following recommendations to help guide research toward more systematic and accurate development:

1. A complementary in situ characterization platform can be established by harnessing the compatibility of various techniques to systematically track interactions between different microregions. Although several in situ electrochemical characterization techniques have been employed to investigate the structural and chemical properties of electrocatalytic interfaces, these experiments are often conducted under different spatiotemporal conditions, making correlations difficult. With advancements in high-resolution X-ray spectroscopy, coupling multiscale in situ spectroscopy techniques offers unprecedented potential to probe the electronic excitations and atomic structures of reaction centers, providing detailed molecular and structural information that single techniques cannot reveal. For example, Ruiter et al. [145] designed an in situ electrochemical cell for X-ray scattering experiments, simultaneously collecting small-angle X-ray scattering and wide-angle X-ray scattering patterns to characterize the morphology and crystal structure of electrocatalysts, respectively. This approach provided real-time insights into structural and morphological changes in electrocatalysts over longer timescales, shedding light on activation, roughening, and deactivation pathways. Moreover, recent studies have demonstrated the effectiveness of coupling X-ray methods with other in situ techniques [146, 147]. Despite the substantial progress enabled by steady-state and quasi-steady-state in situ techniques, a comprehensive mechanistic understanding of CO_2_RR ultimately requires access to short-lived intermediates and transient interfacial processes that occur on ultrafast timescales. Key elementary steps, including initial electron transfer to CO_2_, transient radical formation, rapid changes in metal oxidation states, and early solvation rearrangements, often evolve on femtosecond to microsecond timescales and remain largely invisible to conventional operando measurements. The combination of ultrafast spectroscopies with established operando platforms therefore represents a critical future direction for resolving transient reaction dynamics and constructing a more complete, time-resolved picture of CO_2_RR mechanisms. To facilitate the development of combined multimodal platforms, Table 4 provides a detailed comparison of mainstream in situ techniques. By outlining their complementary strengths and specific limitations, this matrix serves as a strategic guide for integrating multiple methods to achieve a holistic understanding of the CO_2_RR process. Nevertheless, no single technique can fully capture the complex dynamics of the reaction interface. To facilitate the development of combined multimodal platforms, Table 5 provides an applicability matrix that pairs each characterization technique with four microregions, guiding the selection of appropriate technology couplings to achieve cross-microregion characterization combinations that are consistent with specific themes.
2. In addition to coupling in situ techniques, the integration of experimental measurements and theoretical simulations is essential for unraveling CO_2_RR mechanisms. Although in situ spectroscopy can identify reaction intermediates adsorbed on the electrode surface, understanding the reaction kinetics and distinguishing critical intermediates is dependent on atomic-scale insights obtained through numerical calculations. Current DFT calculations often oversimplify interfacial dynamics by focusing solely on the thermodynamic stability of intermediates, neglecting solvent-mediated kinetic effects. The inclusion of solvents is crucial given that water molecules are not passive spectators in the CO_2_RR process but directly participate in the reaction or indirectly influence the cathode–catholyte interface, altering the reaction in either case. This influence includes local dipole field and alkalinity effects induced by cations. Ab initio molecular dynamics (AIMD) simulations offer an effective approach to address these challenges by providing a comprehensive understanding of catalytic structures, interfacial behavior, and solvent effects. However, applying AIMD directly to electrochemical interfaces still faces key challenges. In time and spatial scales, typical AIMD simulations operate only on picosecond to nanosecond and nanometer scales, making it difficult to directly observe rare events (such as C−C coupling) and mesoscale structural evolution. Ultrafast and time-resolved techniques, such as transient absorption spectroscopy, step-scan FTIR, and time-resolved X-ray methods, offer unique opportunities to directly capture these fleeting species and to bridge the gap between theoretical kinetics and experimentally accessible steady-state observables. Future developments require combining AIMD with continuum models, enhanced sampling techniques, and developing more efficient and accurate methods for explicit solvent–electric field coupling calculations to overcome these limitations [148, 149]. Moreover, the vibrational density of states in AIMD explicitly accounts for the anharmonicities of vibrational modes, intramolecular/intermolecular couplings, and solvent dynamics, providing a promising tool to simulate the vibrational dynamics of surface intermediates and forging a link between computational simulations and actual electrochemical spectroscopic measurements [150].
3. Additionally, artificial intelligence (AI)-assisted data analysis based on supervised learning and chemical principles has been systematically developed in materials science and becomes a powerful tool for understanding reaction mechanisms [151]. However, in situ spectroscopic data often presents challenges such as low signal-to-noise ratio, severe signal overlap of intermediates, and the difficulty of extracting weak characteristic signals from complex backgrounds. AI methods, particularly deep learning models, offer new pathways to address these problems. For example, by training convolutional neural networks or denoising autoencoders, one can directly perform noise reduction and feature separation on high-noise in situ Raman or infrared spectra, thereby more accurately identifying potential intermediates [152]. In terms of mechanism analysis, big data analysis using machine learning algorithms revealed that asymmetric coupling mechanisms (*CO–*COH/*CH_2_) exhibited greater potential efficiency compared to symmetric coupling (*CO–*CO) [153], which was consistent with recent in situ characterization results [69]. With increased computational resources and advancements in machine learning algorithms, AI-assisted spectroscopic predictions and data analysis will help to identify additional potential reaction intermediates and elementary steps [154], facilitating the design of catalytic structures with high CO_2_RR selectivity based on precise structure–performance relationships.
4. Furthermore, transitioning in situ techniques from idealized laboratory models to industrial-grade applications remains a formidable challenge. Current characterization tools often face significant operational limitations under high-rate conditions. For example, in situ TEM is restricted by its requirement for ultra-thin samples, which do not accurately represent the complex, thick architecture of industrial GDEs. Meanwhile, infrared spectroscopy and other optical methods are highly susceptible to interference from intense Joule heating and extensive bubble formation at industrial current densities (>500 mA cm⁻^2^). Future efforts should therefore focus on developing more robust and penetrative operando platforms such as high-energy X-ray probes and neutron imaging to visualize mass transport and structural evolution within realistic, high-flux environments. This paradigm shift from to in situ studies will be pivotal in bridging the gap between fundamental mechanism discovery and the deployment of durable, large-scale CO_2_ electrolyzers.

**Table 4:** Capability matrix of in situ techniques for CO_2_RR studies

In situ technique	Primary signal/observable species	Resolution		Compatibility with reactors	Operating window	Limitations
		Temporal	Spatial			
Fourier Transform infrared spectroscopy (FTIR)	Light absorption via electric dipolar interactions: adsorbed intermediates, interfacial water, electrolyte ions	High (ms to s)	Low (1.5–10 mm)	H cell	Full potential windows	Surface sensitivity and limited penetration depth
Raman spectroscopy	Inelastically scattered light via the sample polarizability tensor: surface adsorbates, oxides, carbonates, pH near surface	High (ms to s)	High (~ 250–500 nm)	Flow cell/MEA	Full potential windows	Inherently weak Raman signals; fluorescence interference; local heating
Transmission electron microscopy (TEM)	Transmitted electrons: nanoscale morphology, lattice structure, particle dynamics	High (ms to s)	High (atomic to nm)	Ultrathin liquid cell	Low current density	Beam-induced effects; limited sampling area
Differential electrochemical mass spectrometry (DEMS)	Mass-to-charge ratio: volatile gaseous intermediates and products	High (~ 1s)	None (Sampling-based)	Specialized DEMS setup (H cell)	Fast response; continuous real-time monitoring	Volatile species; interference from background signals
Nuclear magnetic resonance (NMR)	Nuclear spin: molecular structure and chemical environment, liquid products (ethanol, formate), electrolyte environment, water dynamics	Low (min to hr)	None (bulk)	H cell/flow cell	Deuterated solvents	low sensitivity; paramagnetic interference
Electrolysis optical coherence tomography (eOCT)	Light scattering: electrolyte distribution, gas bubble evolution and dynamics, formation and dissolution kinetics of carbonate precipitates	Ultrahigh (< 1 µs)	High (~ 1 µm)	Flow cell/MEA	Highly versatile	Without chemical specificity; penetration depth
Neutron imaging	Neutron attenuation: water distribution with high contrast, gas evolution appears as low-attenuation voids, salt precipitates	Medium (~ 10s)	High (6 µm)	Specific MEA	Highly versatile	Contrast ambiguity
Electron paramagnetic resonance (EPR)	Unpaired electrons: short-lived radical intermediates	Medium (s to min)	None (bulk)	Specialized capillary/flat H cell	Highly versatile	Paramagnetic sensitivity
X-ray absorption spectroscopy (XAS)	X-ray absorption coefficient: XANES: oxidation state, coordination, orbital occupancy; EXAFS: coordination, bond length	Medium (mins)	None (bulk)	H cell/flow cell/ MEA	Highly versatile	Low time resolution
Small-/wide-Angle X-ray scattering (SAXS/WAXS)	X-ray scattering intensity: SAXS: particle size distribution, micropore information WAXS: atomic-scale crystal structure parameters, phase composition	High (ms to s)	High (SAXS: ~ 1–100 nm; WAXS: ~ 0.1–100 nm)	Flow cell/MEA	Highly versatile	Lack of chemical specificity; Spatial sensitivity
X-ray diffraction (XRD)	Bragg diffraction: crystal structure, phase transitions, crystallinity	Medium (s to min)	None (bulk)	H cell/flow cell	Highly versatile	Spatial sensitivity; potential beam effects
X-ray photoelectron spectroscopy (XPS)	Photoelectrons: surface oxidation states, elemental composition, electronic structures	Low (mins)	Medium (~ 200 µm)	Vacuum-compatible H cell	Low pressure and high vacuum constraints	High vacuum conditions

^*^Time and spatial resolutions are indicative and depend on specific cell design and data acquisition strategies

**Table 5:** Applicability matrix of in situ characterization techniques across the four distinct microregions in CO_2_RR systems

In situ technique	Electrolyte environment	Reaction interface	Catalyst structure	Diffusion field
FTIR	●	●	×	×
Raman spectroscopy	●	●	○	×
TEM	×	○	●	×
DEMS	×	●	×	×
NMR	●	○	×	○
eOCT	×	×	×	●
Neutron imaging	×	×	×	●
EPR	×	●	●	×
XAS	×	○	●	×
SAXS/WAXS	×	○	●	×
XRD	×	×	●	×
XPS	×	●	●	×

● — Highly available; ○ — Limited; × — Unavailable

In summary, establishing more accurate structure–activity relationship requires not only strengthening the understanding of each microregion but also establishing systematic connections between microregions. Forming such connections will rely on advanced in situ technologies and their integration with theoretical methods. Therefore, we call for greater participation of optoelectronic physics and theoretical computational chemistry in building multiscale characterization platforms and developing high-precision numerical simulations. This synergistic approach will open new opportunities for achieving sustainable energy storage and conversion.
